# Temperature-Dependent Kinetics of the Methyl Vinyl Ketone Oxide Criegee Intermediate: Self-Reaction and Reaction with Trifluoroacetic Acid

**DOI:** 10.1021/acs.jpca.6c01738

**Published:** 2026-05-27

**Authors:** Saurabh Khodia, Eddie Reilly, Maria de los Angeles Garavagno, Andrew J. Orr-Ewing

**Affiliations:** School of Chemistry, 1980 University of Bristol, Cantock’s Close, Bristol BS8 1TS, U.K.

## Abstract

Direct kinetic measurements are reported for the self-reaction and unimolecular decay of methyl vinyl ketone oxide (MVKOO), and its reactions with trifluoroacetic acid (TFA) and formic acid (FA). The *syn*-MVKOO stabilized Criegee intermediate was generated by laser flash photolysis of 1,3-diiodobut-2-ene in excess O_2_ and monitored via its absorption at 360 nm using cavity ring-down spectroscopy. Time-resolved MVKOO decay traces recorded in the absence of added coreactants were analyzed to separate the first-order unimolecular thermal decomposition from second-order self-reaction contributions. Within the 270–330 K temperature and 40–200 Torr pressure ranges studied, MVKOO undergoes rapid second-order self-reaction with a rate coefficient *k*_SR_ = (12 ± 4) × 10^–10^ cm^3^ s^–1^ that shows no significant temperature or pressure dependence. At 292 K, the unimolecular decay rate coefficient is *k*_uni_ = (50  ±  21) s^–1^, averaged over 40–200 Torr measurements, and shows a positive temperature dependence. Bimolecular reactions of MVKOO with FA and TFA were investigated under pseudo-first-order conditions, yielding rate coefficients of *k*_FA_ = (1.9 ± 0.2) × 10^–10^ cm^3^ s^–1^ and *k*_TFA_ = (3.8 ± 0.3) × 10^–10^ cm^3^ s^–1^ at 292 K, respectively. The MVKOO + TFA reaction exhibits a weak negative temperature dependence. The measured rate coefficients for MVKOO reaction with FA and TFA are consistent with the predictions from a structure–activity relationship based on dipole-mediated interactions. These findings quantify key unimolecular and bimolecular loss processes of MVKOO and further highlight the potential atmospheric significance of Criegee intermediate–acid chemistry in organic oxidation pathways.

## Graphical abstract

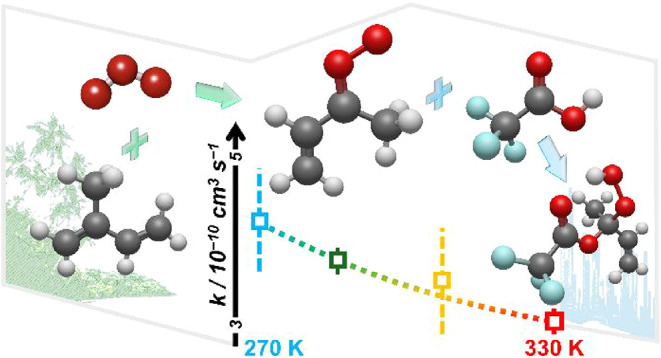

## Introduction

The ozonolysis of unsaturated volatile organic compounds (VOCs) represents a key source of stabilized Criegee intermediates (SCIs) in the troposphere, which can potentially impact atmospheric oxidation chemistry.^1,2^ Among biogenic VOCs, isoprene (2-methyl-1,3-butadiene) is the most abundant atmospheric alkene and contributes substantially (∼10%) to the formation of secondary oxidation products through reaction with ozone.^3,4^ Ozone addition across the double bonds of isoprene produces a range of substituted SCIs, whose subsequent unimolecular decay and bimolecular reactions influence OH radical budgets, atmospheric trace gas lifetimes, and secondary organic aerosol (SOA) formation.^3^ Understanding the kinetics and fate of individual isoprene-derived SCIs is therefore essential for constraining atmospheric chemical models. Methyl vinyl ketone oxide (MVKOO, (CH_2_CH)­(CH_3_)­COO) is a four-carbon SCI formed predominantly via isoprene ozonolysis and is estimated to account for ∼11–23% of the SCI yield from this reaction, together with 58% formaldehyde oxide (CH_2_OO) and 19% methacrolein oxide under tropospheric conditions.^3,5^ We adopt the abbreviation MVKOO following the Master Chemical Mechanism (MCM),^6^ which aligns with the related notation MVK-OO used by Wennberg and co-workers^3^ and by Lester and co-workers.^7^ In contrast to the simplest SCI CH_2_OO, MVKOO contains an unsaturated vinyl substituent conjugated with the carbonyl oxide functional group, resulting in extended π-electron delocalization and enhanced resonance stabilization.^7^ This structural complexity fundamentally distinguishes MVKOO from smaller SCIs by altering its electronic properties, conformational flexibility, and reaction pathways.^7–11^ As a consequence, MVKOO may exhibit chemical behavior that is not adequately captured by extrapolation from CH_2_OO kinetics alone, despite the latter often being used as a benchmark system.

One of the most significant differences between MVKOO and CH_2_OO lies in their contrasting reactivities toward gaseous water and water dimers. The reaction of CH_2_OO with water vapor, particularly via water dimers with a rate coefficient of (6.5 ± 0.8) × 10^–12^ cm^3^ s^–1^, constitutes the dominant atmospheric sink for this SCI under most tropospheric conditions, limiting the availability of CH_2_OO for reactions with other trace gases.^12^ By contrast, MVKOO exhibits substantially slower reactions with water molecules (*k* < 4.0 × 10^–17^ cm^3^ s^–1^) and with water dimers (*k* < 3.0 × 10^–14^ cm^3^ s^–1^).^8^ This reduced reactivity is a consequence of a higher transition state (TS) barrier arising from the disruption of conjugation in MVKOO due to steric bending of the carbonyl oxide out of plane in the MVKOO–water TS complex.^8^ The relatively slow reactivity of MVKOO with water vapor enables reactions with other atmospheric molecules to compete even under humid conditions, thereby enhancing its atmospheric significance.

MVKOO has four conformational isomers resulting from rotation around the carbonyl oxide bond and the orientation of the C=C and C=O double bonds. *Syn*- and *anti*- describe the methyl group orientation relative to the terminal oxygen, while *cis* and *trans* describe the relative positions of the two double bonds.^7^ The *cis*-*trans* isomerization is calculated to be rapid under thermal conditions,^8,13^ leading to the preferential formation of the more stable *trans* isomer. The *syn-anti* interconversion is substantially less feasible, as rotation about the C=O bond entails a much higher energy barrier (>100 kJ mol^–1^).^7^ Experimentally, the *syn*-*trans* conformer was identified to dominate the thermal population at a temperature of 298 K and pressures ranging from 4 to 700 Torr N_2_.^8,14^ In *syn*-*trans*-MVKOO, the methyl substituent is oriented in the same direction as the terminal oxygen atom of the carbonyl oxide and there is a *trans* arrangement of the vinyl group with respect to the carbonyl. No *anti*- conformers were isolated experimentally, consistent with the theoretical prediction of rapid unimolecular decomposition of *anti*-MVKOO. Specifically, *anti*-MVKOO undergoes cyclic isomerization to the dioxole with rates 2–3 orders of magnitude faster than the 1,4-hydrogen-atom transfer pathway observed for *syn*-MVKOO.^7,14,15^

Lester and co-workers demonstrated the selective synthesis of MVKOO via 248 nm photolysis of a 1,3-diiodo-but-2-ene precursor in the presence of excess molecular oxygen.^7^ In this approach, photolysis produces a monoiodoalkene radical that rapidly reacts with O_2_ to form MVKOO under collisionally stabilized conditions. This method enabled the first time-resolved spectroscopic and kinetic studies of MVKOO. Detection is typically achieved via ultraviolet (UV) absorption near 350–360 nm, which is particularly sensitive to the *syn-trans* conformer.^8,11^ The UV electronic spectrum of MVKOO has been characterized experimentally and interpreted with the aid of high-level electronic structure calculations.^8,11^ The observed broad absorption band in the 300–450 nm region is assigned primarily to a π* ← π electronic transition that promotes molecules to a repulsive region of the excited state, leading to dissociation of the O–O bond in MVKOO.^11^

Bimolecular reactions of substituted SCIs are often governed by long-range electrostatic interactions and the formation of prereactive complexes.^2,16,17^ Reactions between stabilized Criegee intermediates and organic acids have attracted considerable attention due to their potential role in rapid oligomerization chemistry and SOA formation.^18–20^ These reactions proceed at near-collision-limited rates and yield low-volatility products. Structure–activity relationship (SAR) models based on dipole–dipole capture theory have successfully rationalized the large rate coefficients observed for reactions of some of the smaller SCIs with organic acids across a range of systems.^16,17^ However, analogous reactions involving larger, more substituted SCIs such as MVKOO remain comparatively unexplored. Trifluoroacetic acid (TFA) is a persistent atmospheric carboxylic acid arising primarily from the degradation of fluorinated compounds such as hydrofluoroolefins,^21^ and exhibits distinct reactivity due to its strongly electron-withdrawing CF_3_ group.^16,22^ Given the enhanced stability of MVKOO under humid conditions, its reaction with TFA represents an environmentally relevant example of SCI-driven acid chemistry in fluorinated systems.

Here, we report direct measurements of the rates of unimolecular decay of MVKOO, its bimolecular self-reaction, and its reactions with formic acid (FA) and TFA. The time-resolved concentration of MVKOO is monitored using its well-characterized ultraviolet–visible (UV–vis) absorption spectrum in a flash photolysis experiment coupled with cavity ring-down spectroscopy (CRDS).^23^ The reactivity of MVKOO is compared with that of the simpler SCI, CH_2_OO, to assess the impact of molecular complexity on Criegee intermediate-acid chemistry. Compared with the extensively studied smaller Criegee intermediates, experimental bimolecular kinetic data for MVKOO remain limited and are available only for a small number of atmospheric reactants.^8,24–27^ Further studies are therefore necessary to establish reliable structure–activity relationships for larger atmospherically relevant substituted Criegee intermediates. The measured rate coefficients are evaluated in the context of the dipole-capture SAR model and a prereaction complex kinetic model to probe the role of long-range electrostatic interactions in governing the reaction dynamics.^17^ By examining these reactions under atmospherically relevant temperature conditions, this study provides kinetic data needed to evaluate the potential role of substituted Criegee intermediate reactions with organic acids in pathways leading to oligomer formation and secondary organic aerosol production.

## Materials and Methods

The *Z*/*E*-1,3-diiodobut-2-ene precursor for MVKOO generation was synthesized following a modified version of the procedure reported by Barber et al.^7^ Briefly, but-2-yn-1-ol (99% Sigma-Aldrich) was reacted with trimethylsilyl iodide (99% Sigma-Aldrich) in dry diethyl ether at −40 °C, followed by warming to room temperature, aqueous quenching, and purification by silica gel chromatography to afford a *Z*/*E* mixture of 1,3-diiodobut-2-ene. Additional chromatographic purification yielded a yellow oil product. NMR (Figure S1) and UV–vis absorption spectroscopy (Figure S2) measurements confirmed the high sample purity.

In the present work, MVKOO was generated *in situ* following the method developed by Lester and co-workers, in which a *Z*/*E* mixture of the 1,3-diiodobut-2-ene vapor was photolyzed in the presence of excess molecular oxygen.^7^ Unlike the original method, which employed 248 nm photolysis, 1,3-diiodobut-2-ene was photolyzed at 266 nm (30 mJ per pulse, fluence ∼150 mJ cm^–2^, <10 ns pulse duration) using the fourth-harmonic output of a Continuum Surelite SLI-10 Nd:YAG laser.^7^ The diiodo precursor absorbs sufficiently at 266 nm, with an absorption cross section σ_266 nm_ = 8.6 × 10^–18^ cm^2^,^25^ to enable efficient photolysis. The resulting MVKOO concentration was monitored by CRDS at a probe wavelength of 360 nm, generated by frequency doubling the output of a tunable dye laser (Sirah CSTR-LGA-24) operating on pyridine 2 dye pumped by the second harmonic of an Nd:YAG laser (Continuum Surelite SLIII-10). This probe wavelength overlapped the strong and broad π* ← π electronic absorption band of MVKOO.

The kinetics of MVKOO unimolecular decay and bimolecular reactions were determined by systematically varying the temporal delay between probe and photolysis laser pulses. A 2 Hz laser repetition rate was used to ensure each pulse probed a fresh portion of the flowing sample gas mixture, eliminating contributions from prior photolysis events. These experiments were conducted in a flow reactor that was described in detail previously.^23^ The reactor comprised a double-jacketed glass flow cell integrated with a 106 cm long optical cavity for CRDS. The reagents and bath gases were introduced into the glass flow tube along the detection axis at controlled flow rates using mass flow controllers (MFC). Temperature control was achieved by circulating a refrigerant through the inner jacket using a Huber Unistat 360 heating–cooling unit, while the outer jacket was kept under dry air to provide thermal insulation. High-purity molecular nitrogen (N_2_) and oxygen (O_2_) were used as bath gases. Varied flow rates of N_2_ were employed for pressure-dependent studies.

The *Z*/*E*-1,3-diiodobut-2-ene (synthesized), FA (>99% Sigma-Aldrich), and TFA (>99% Thermo Fisher) samples were further purified by multiple freeze–pump–thaw cycles. Diluted samples of *Z*/*E*-1,3-diiodobut-2-ene (0.03/750 Torr in N_2_), FA (0.5/750 Torr in N_2_) and TFA (0.7/750 Torr in N_2_) were prepared by premixing the pure sample vapors with N_2_ at known dilution ratios in separate glass bulbs, followed by overnight equilibration to ensure complete homogenization. The glass bulbs were blacked out to protect the samples from ambient light during preparation and storage. The bimolecular kinetics experiments were performed at a minimum total pressure of 50 Torr, as the vapor pressure of the MVKOO precursor *Z*/*E*-1,3-diiodobut-2-ene at room temperature is low (<0.05 Torr at 20 °C). The chosen conditions ensured adequate precursor concentration for photolysis in the flow reactor, producing MVKOO at concentrations suitable for reliable kinetic measurements with good signal-to-noise ratios. The initial MVKOO concentration was typically maintained at about 4 × 10^11^ cm^–3^, as determined using the reported thermalized absorption cross section.^13^ The details of the concentration estimation for MVKOO are further discussed below. The TFA concentration was varied within the 10^13^ cm^–3^ range, maintaining an excess compared to MVKOO to ensure conditions for pseudo-first-order kinetics.

## Results and Discussion

Figure 1 shows the decay of the MVKOO absorption in the absence of any coreactant. The *x*-axis represents the delay time of the probe laser with respect to the photolysis pulses. The photolysis laser pulse initiates the reaction (*t* = 0) by photodissociating 1,3-diiodobut-2-ene in the presence of excess O_2_, leading to the formation of MVKOO. The probe laser is then introduced at defined delay time intervals after photolysis to measure the time-dependent MVKOO concentration. The MVKOO signal rises on a submillisecond time scale after the photoinitiation of the reaction. This rapid onset reflects the swift conversion of photoexcited diiodo precursors by C–I bond cleavage and reaction with O_2_, which is present in large excess under our conditions ([O_2_] ∼ 3 × 10^17^ cm^–3^ vs diiodo-precursor ∼3 × 10^13^ cm^–3^). The observed time scale of MVKOO formation (100–200 μs) is consistent with the reported bimolecular rate coefficient for the monoiodoalkene radical, (^•^C_4_H_6_I) + O_2_ reaction (*k* = (1.7 ± 0.1) × 10^–13^ cm^3^ s^–1^).^8^

**Figure 1:**
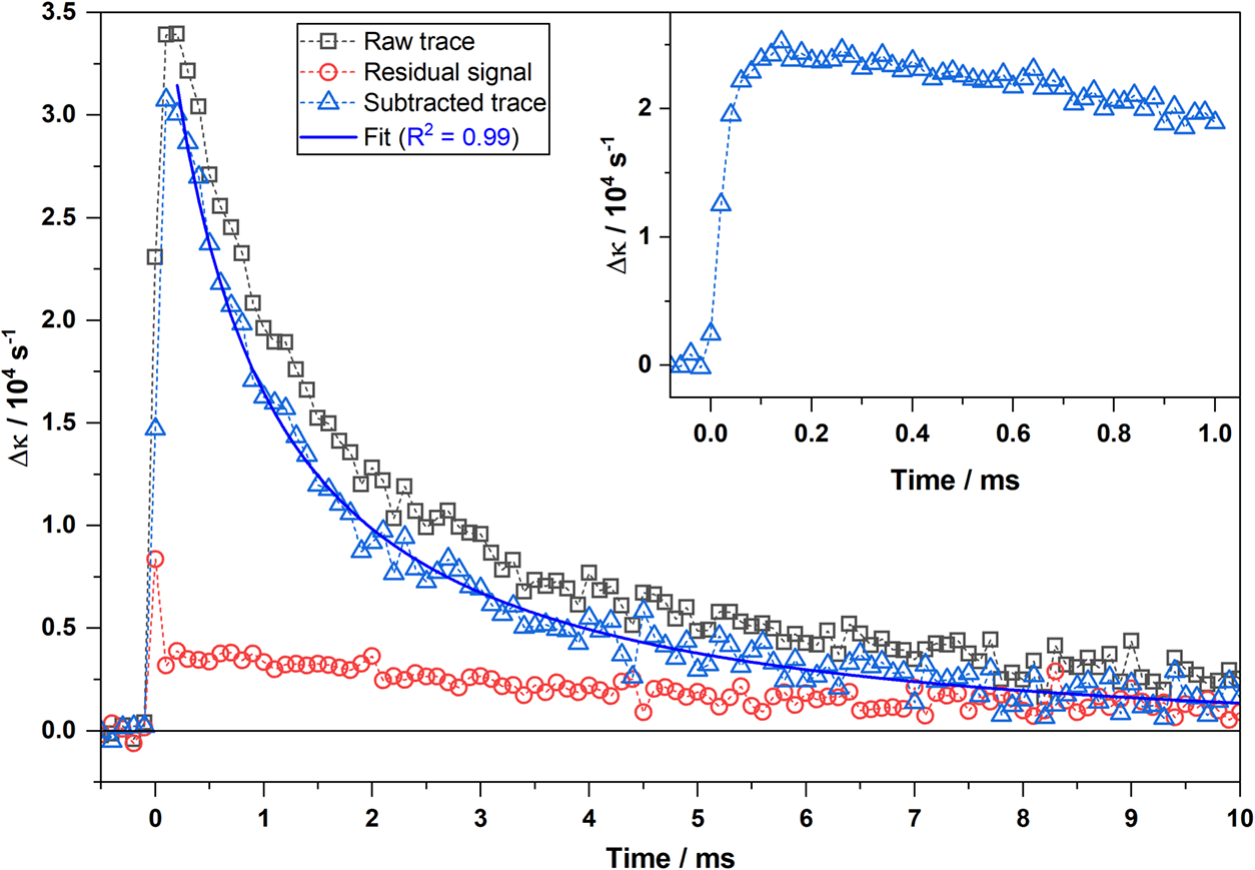
Decay trace of MVKOO monitored at the 360 nm CRDS probe wavelength. Black squares represent the raw signal in the absence of a coreactant. A byproduct formed during MVKOO generation contributes to absorption at the probe wavelength. Red circles show the signal obtained in the presence of excess TFA (>5 × 10^14^ cm^–3^), which selectively scavenges MVKOO and isolates the residual interference signal. Blue triangles denote the MVKOO decay corrected for the residual interference signal, obtained by subtracting the red circles from the black squares. The solid blue line is a fit to a kinetic expression (E1) describing simultaneous first- and second-order loss of MVKOO. The fit gives first- and second-order rate components of (138 ± 16) s^–1^ and (3.2 ± 0.2) × 10^7^ cm s^–1^, respectively, the latter scaled by the MVKOO absorption cross section at 360 nm. The inset shows the early time decay on a 1 ms time scale with an initial MVKOO concentration of ∼3 × 10^11^ cm^–3^. The experiment was conducted at 50 Torr and 298 K.

The vertical *y*-axis shows the change in the cavity ring-down rate Δκ (s^–1^), which is directly proportional to the MVKOO concentration, *N*(*t*) = (Δκ × *L*)/(*c* × *d* × σ_360 nm_), where *L* = 106 cm is the cavity length, *d* = 7.6 cm is the overlap length between the photolysis and probe laser beams, *c* is the speed of light, and σ_360 nm_ is the absorption cross section of MVKOO at the 360 nm probe wavelength (σ_360 nm_ = (3.4 ± 0.7) × 10^–17^ cm^2^).^13^ In our experimental measurements, the quantity Δκ is obtained as Δκ = (1/τ_on_) – (1/τ_off_), where τ_on_ and τ_off_ are the ring-down times with the photolysis laser on and off, respectively. For an empty cavity, a typical ring-down time of 6 μs is observed, which is much shorter than the 0.5–10 ms reaction time scale, ensuring the MVKOO loss kinetics are fully resolved. The diiodo-precursor does not significantly affect the baseline Δκ values as it does not absorb at the CRDS probe wavelength of 360 nm.^25^ However, minor absorption from species other than MVKOO contributes to the observed signal, as discussed below. These contributions were accounted for in the analysis to ensure that the absorbance measured at 360 nm reflects the true MVKOO concentration.

MVKOO generation from the photoexcited 1,3-diiodobut-2-ene precursor proceeds via rapid formation of the monoiodoalkene radical and subsequent iodine-atom substitution by O_2_ (Scheme 1).^14,27^ Although not directly evident in the raw MVKOO decay trace (Figure 1), the formation of byproducts during MVKOO generation, as depicted in Scheme 1, including iodine monoxide (IO, σ_360 nm_ ∼ 1.9 × 10^–18^ cm^2^),^28^ methyl vinyl ketone (MVK, σ_360 nm_ ∼ 3.6 × 10^–20^ cm^2^),^29^ and IMVKOO (CH_3_(C_2_H_3_)­CIOO) adduct, is expected to contribute an interfering absorption at 360 nm.^30^ If the 266 nm photoexcited precursor molecules have sufficient internal energy to eliminate two I atoms, one further possible source of interference is biperoxy radicals with peroxy groups on different carbon atoms formed by prompt reaction of the resulting biradical intermediates with the excess O_2_. This interference is expected to be less significant here than in the prior experiments using 248 nm excitation. To account for these sources of interference, decay traces were recorded in the presence of excess TFA, which selectively and rapidly scavenges MVKOO while leaving the residual signal largely unaffected. In Figure 1, the black squares represent the raw decay trace of MVKOO in the absence of coreactant, while the red circles show the signal obtained in the presence of excess TFA (>5 × 10^14^ cm^–3^), which scavenges MVKOO and isolates the residual contribution from other absorbing species independent of TFA concentration. Subtracting the TFA-scavenged trace from the raw signal yields the decay of MVKOO corrected for contributions from other absorbing species (blue triangles). This approach was applied consistently in all subsequent measurements, including bimolecular reactions, to correct for the persistent residual interference signal and ensure that the observed decay reflects the true MVKOO concentration (Figure S3).

**Scheme 1:**
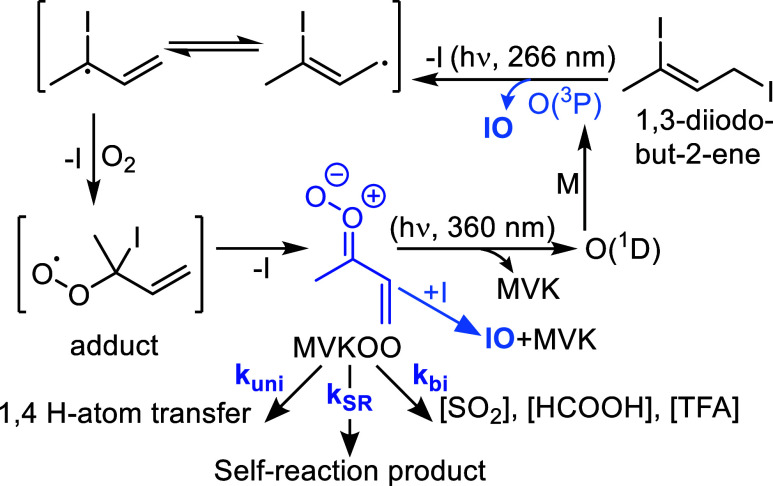
Schematic Representation of Formation and Decay Pathways for MVKOO

As is noted above, several species formed during MVKOO generation might contribute to absorption at the 360 nm probe wavelength. The iodoperoxy adduct IMVKOO is expected to contribute at early times because it forms directly from the reaction of the photolytically generated radical with O_2_ before dissociation to MVKOO. In contrast, IO and other possible branching products arise from secondary chemistry involving MVKOO (as illustrated in Scheme 1) and are therefore expected to appear at later times. In the presence of excess TFA, MVKOO reacts rapidly with the acid and should therefore be removed much faster than it can react with the lower concentration of I atoms, thereby strongly suppressing IO formation. The persistence of the residual signal at later times and its rapid rise even in the presence of excess TFA suggest that additional absorbing species or pathways may contribute. Possible formation routes for species absorbing at the probe wavelength were therefore examined and are discussed in detail in the Supporting Information.

The background-corrected decay trace was then fitted using eq E1 to account for simultaneous first- and second-order (self-reaction) loss of MVKOO, d­[MVKOO]/d*t* = −*k*_uni_[MVKOO] – 2*k*_SR_[MVKOO]^2^, where *k*_uni_ and *k*_SR_ are first- and second-order rate coefficients, respectively. The factor of 2 in the second-order term accounts for the bimolecular self-reaction of MVKOO. The integrated solution to this differential equation expressed in terms of Δκ is given as^23,31^

E1 $$\Delta \kappa \left(t\right)=\frac{k_{\mathrm{uni}}}{\frac{k_{\mathrm{uni}}}{\Delta \kappa \left(t_{0}\right)}e^{k_{\mathrm{uni}}t}-{k^{\prime }}_{\mathrm{SR}}\left(\frac{2L}{\mathrm{cd}}\right)+{k^{\prime }}_{\mathrm{SR}}\left(\frac{2L}{\mathrm{cd}}\right)e^{k_{\mathrm{uni}}t}}$$

Here, *k*_uni_ accounts for all first-order and pseudo-first-order loss processes, while *k*′_SR_ represents the second-order rate coefficient for the self-reaction of MVKOO scaled by the MVKOO absorption cross-section at the probe wavelength (*k*′_SR_ = *k*_SR_/σ_360 nm_). Using the initial Δκ­(*t*_0_) value obtained from the fit (solid blue line, Figure 1), combined with the reported absolute absorption cross section of thermalized MVKOO at 360 nm, an initial MVKOO concentration of ∼4 × 10^11^ cm^–3^ is estimated.

### Self-Reaction of MVKOO

In the absence of a coreactant, kinetic measurements such as those discussed above are consistent with MVKOO loss having both unimolecular decay and self-reaction contributions. As shown in Figure 1, the concentration decreases to less than 10% of its initial value within 10 ms. Fitting the decay trace with eq E1, which includes both first- and second-order terms, provides an excellent fit (*adj*. *R* ^2^ = 0.99). An equally good fit (*adj*. *R* ^2^ = 0.98) is obtained using the second-order expression alone, indicating that the first-order contribution is minor relative to the dominant self-reaction pathway under our experimental conditions of MVKOO concentration.

Even without an added coreactant, several competing loss pathways can operate concurrently in addition to the rapid second-order self-reaction loss, including first-order loss via unimolecular decay, reaction with side products like IMVKOO, I or IO, wall losses, and diffusion or mass flow out of the probe laser volume. These processes collectively govern the effective lifetime of MVKOO in the flow reactor and must be accounted for to interpret the subsequent bimolecular kinetics accurately. The physical loss processes, such as at the reactor walls or by diffusion and mass flow, were previously characterized for our experimental setup^16,23^ and are negligible within the uncertainty of the measurement. Moreover, the fitted *k*_uni_ value is consistent with the reported unimolecular decay rate of MVKOO,^14^ so the overall first-order component is attributed predominantly to unimolecular loss of MVKOO (*k*_uni_), although minor contributions from reactions with iodine-containing species (e.g., IMVKOO, I, or IO) cannot be excluded. Decay measurements were performed over a total pressure range of 40–200 Torr at 292 K by varying the N_2_ bath gas flow while maintaining constant concentrations of 1,3-diiodobut-2-ene and O_2_. As shown in Figure S4, neither the self-reaction rate coefficient (*k’*_SR_) scaled by the absorption cross section, nor the unimolecular loss (*k*_uni_) component shows a measurable pressure dependence within experimental uncertainty.

The lack of pressure dependence for both *k’*_SR_ and *k*_uni_ indicates that these pathways proceed independently of third-body effects under our experimental conditions. In the present study, MVKOO formation is effectively instantaneous (∼200 μs) across the entire 40–200 Torr range, with no observable pressure-induced retardation. This behavior differs from that reported by Lin et al., who observed delayed MVKOO formation (∼1–2 ms) at pressures above 50 Torr and attributed it to collisional stabilization of the IMVKOO adduct prior to its dissociation to MVKOO. In our experiments, MVKOO appears rapidly and reaches a maximum at early times, followed by decay. This difference likely reflects the higher MVKOO concentrations used here, which shift the onset of the MVKOO self-reaction to earlier times, as illustrated in Figure S5, where increasing the MVKOO concentration shifts the signal maximum from ∼1 ms to ∼0.1 ms. Importantly, the MVKOO concentration was kept constant across all pressure-dependent measurements. Experiments were conducted at high flow rates with highly diluted precursor (<0.002% of the total gas), ensuring rapid renewal of the reaction mixture in the flow reactor and minimizing secondary chemistry from accumulated reactive species.

The effect of the 1,3-diiodobut-2-ene precursor concentration on MVKOO kinetics was investigated by varying its initial concentration from 8 × 10^12^ to 4 × 10^13^ cm^–3^, thereby changing the initial MVKOO concentration and its kinetic profile. Both the scaled self-reaction (*k*′_SR_) and unimolecular loss (*k*_uni_) rate coefficient values showed no significant dependence on the precursor concentration (Figure S5). MVKOO formation occurred with an effective yield of ∼2% relative to the precursor concentration (Table S1). At higher MVKOO concentrations, the initial decay is dominated by the self-reaction, which can prevent the fit function E1 from isolating the slower unimolecular component. However, the determination of *k*′_SR_ is not affected. The self-reaction rates measured in multiple experiments (see Table S1) give an average value of *k*′_SR_ = (3.6 ± 0.8) × 10^7^ cm s^–1^. The corresponding unscaled self-reaction rate coefficient (*k*_SR_) for MVKOO was determined by multiplying the measured *k*′_SR_ by the absorption cross section at 360 nm, giving *k*_SR_ = (12 ± 4) × 10^–10^ cm^3^ s^–1^. This value indicates that MVKOO undergoes rapid self-reaction.

The dipole moments of MVKOO, (CH_3_)_2_COO and CH_2_OO were computed at the DFT-D3­(BJ)/B3LYP/aug-cc-pVTZ level. For MVKOO, values of 5.27 D (*syn*–*trans*), 5.01 D (*syn*–*cis*), 5.64 D (*anti*–*trans*), and 4.88 D (*anti*–*cis*) were obtained. The dipole moment of the most stable *syn*–*trans* conformer (5.27 D) was used for kinetic analysis. This MVKOO dipole moment value is comparable to that of (CH_3_)_2_COO (5.42 D) and larger than that of CH_2_OO (4.39 D), indicating similarly strong long-range electrostatic interactions. The collisional and dipole capture limits were calculated for MVKOO using the expressions reported by Chhantyal-Pun et al.^31^ Relative to (CH_3_)_2_COO, the larger effective diameter of MVKOO increases the hard-sphere collisional kinetic limit by approximately a factor of 1.6, whereas the dipole capture limit is slightly reduced due to its marginally smaller dipole moment and greater mass. The experimentally determined self-reaction rate coefficient *k*_SR_ = (12 ± 4) × 10^–10^ cm^3^ s^–1^, exceeds the estimated collisional limit but remains below the dipole capture limit (Table S2). This observation is consistent with the steric and orientational constraints expected from the vinyl group substitution in MVKOO.

Despite its rapid kinetics, the self-reaction of MVKOO does not represent a significant atmospheric sink because Criegee intermediate concentrations in the troposphere are expected to be very low (∼10^4±1^ cm^–3^).^32^ In contrast, under laboratory conditions where elevated MVKOO concentrations are often generated, self-reaction can become competitive and must be properly quantified. For collisionally stabilized MVKOO in the atmosphere, reactions with water and water dimer are reported to be slow compared to other Criegee intermediates such as CH_2_OO or CH_3_CHOO.^8^ Consequently, unimolecular decay will likely be an important loss pathway for MVKOO under atmospheric conditions. The following section explores the unimolecular decay kinetics and examines their temperature dependence.

### Unimolecular Kinetics of MVKOO

Under our experimental conditions, the unimolecular decay of MVKOO is expected to be dominated by the *syn* conformer, as *cis* and *trans* forms rapidly interconvert under thermalized conditions.^8,14^ The *anti*-MVKOO is predicted to decompose more rapidly, with calculated unimolecular rates spanning ∼2 × 10^3^ to 6.4 × 10^4^ s^–1^,^7,8,33,34^ so even if formed at low yield, it would decay within a few hundred microseconds and not contribute to the observed kinetics. Pressure-dependent CRDS measurements of MVKOO decay at 292 K in the absence of coreactants (Figure S4) yield an average unimolecular loss rate of *k*_uni_ = (50  ± 21) s^–1^. This value sits between published calculated master equation thermal unimolecular loss predictions (33 s^–1^ at 298 K)^7^ and an experimental estimate from Lin et al. for the *syn*-conformer (70 ± 15 s^–1^, at 298 K).^14^ Note that the self-reaction of MVKOO is well described by a single second-order component, with no evidence of additional contributions from other conformers, further supporting that *syn*-MVKOO dominates under our conditions.

The first-order loss is driven by a previously characterized 1,4-hydrogen atom transfer mechanism for *syn*-MVKOO that leads to OH radical formation.^7,15^ Although reactions involving I or IO could in principle contribute under our experimental conditions in which the precursor is in large excess over MVKOO, any significant iodine-mediated or precursor-mediated contribution would be expected to produce a systematic dependence of *k*_uni_ on precursor concentration. No such dependence is observed (Table S1) and the extracted *k*_uni_ values are consistent with previous reports.^7,13^ This analysis indicates that iodine-mediated contributions are negligible within our experimental uncertainty and that the first-order loss is dominated by unimolecular decay of MVKOO. The temperature dependence of *k*_uni_ values was explored at 50 Torr total pressure with varying temperature from 273 to 323 K (Figure 2). A strong temperature dependence is observed. The Arrhenius fit to temperature-dependent *k*_uni_ values (Figure 2 top-inset) gives an effective activation energy *E*_a_ = 24 ± 3 kJ mol^–1^, which is ∼30% lower than the previously reported value, but it still lies within the experimental uncertainty of the earlier measurement.^14^ No significant change is observed in the self-reaction rate coefficient (Figure 2), indicating that *k*′_SR_ is effectively independent of temperature over the explored range.

**Figure 2:**
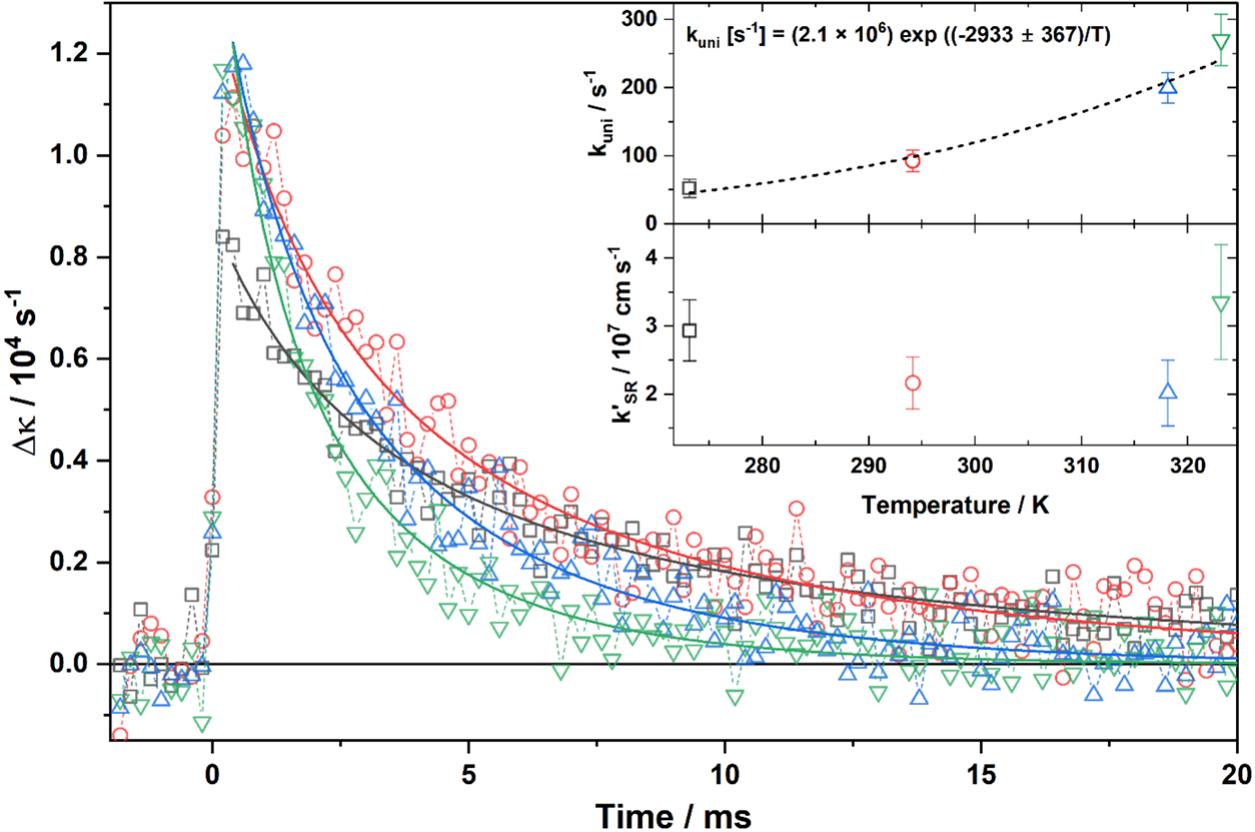
Temperature-dependent first-order and second-order kinetics of MVKOO in the absence of coreactant. The inset shows self-reaction k′_SR_ and unimolecular *k*_uni_ rate coefficients with 1σ uncertainty from the fit function E1 applied to the MVKOO decay trace. All measurements were made at 50 Torr. The dashed line in the top inset represents an Arrhenius fit to temperature-dependent *k*_uni_.

In comparison with other Criegee intermediates, MVKOO exhibits faster unimolecular decay (50 ± 21 s^–1^, at 292 K) than CH_2_OO (11 s^–1^, at 298 K) but remains substantially slower than (CH_3_)_2_COO, which has an approximately six times faster unimolecular loss (Table S2).^23,31^ This trend highlights the strong structural dependence of Criegee intermediate unimolecular kinetics, with faster decay observed for species where internal H-shift pathways are more accessible.

### Bimolecular Kinetics of MVKOO Reactions

The temperature- and pressure-dependent rate coefficients for the reaction of MVKOO with TFA were investigated as a representative example of stabilized Criegee intermediate reactions with halogenated acids. MVKOO reactivity has previously been explored with various atmospheric species, including SO_2_, water, formic acid, dimethyl sulfide, nitric acid and 3-aminopropanol.^8,24–27^ Among these, reactions with acids are relatively fast due to the submerged barrier along the 1,4-insertion pathway.^8^ Prior to studying the reaction with TFA, validation experiments were carried out using MVKOO reactions with SO_2_ and FA (HC­(O)­OH) to verify consistency with previously reported rate coefficients.

The measured rate coefficient for MVKOO reaction with SO_2_, (4.0 ± 0.2) × 10^–11^ cm^3^ s^–1^ (at 50 Torr and 294 K, Figure S6), agrees well with the previously reported value (3.9 ± 0.5) × 10^–11^ cm^3^ s^–1^.^8^ We note that, at higher SO_2_ concentrations, significant interfering absorption was observed from the SO_2_/N_2_/O_2_ mixture itself in the flow reactor. This interference is likely due to photolysis of SO_2_ at the 266 nm wavelength, producing byproducts that absorb at the 360 nm probe wavelength. No such interference was observed for organic acids under the same conditions. The measured rate coefficient for the MVKOO + FA reaction, (1.9 ± 0.2) × 10^–10^ cm^3^ s^–1^ (at 50 Torr and 294 K, Figure S7), is slightly lower than a previously published value of (3.0 ± 0.1) × 10^–10^ cm^3^ s^–1^.^8^ This small deviation may arise from differences in experimental conditions, such as temperature, pressure, and bath gas, as well as from the explicit accounting of first- and second-order losses. In our analysis, the intercept from the linear fit of the *k*_pseudo_ vs FA concentration plot (∼30 s^–1^) was unconstrained and consistent with the first-order unimolecular decay of MVKOO, although its uncertainty is large relative to the *k*_pseudo_ values. Details on how unimolecular and self-reaction losses are accounted for in the bimolecular kinetics analysis are provided below. Overall, the satisfactory agreement with published rate coefficients validates the experimental approach and provides confidence to proceed with the investigation of the MVKOO reaction of interest.

Figure 3 shows decay traces for MVKOO recorded at different TFA concentrations. A large excess of TFA was maintained relative to MVKOO to establish pseudo-first-order conditions. The decay traces were fitted with the fit function E1 as described earlier to account for simultaneous first- and second-order loss processes, with the first-order rate coefficient *k*_uni_ in E1 replaced by *k*_pseudo_. In the fits, the second-order self-reaction rate coefficient, *k*′_SR_ was fixed to a value of 3.6 × 10^7^ cm s^–1^ obtained for MVKOO in the absence of any coreactant, while *k*_pseudo_ was allowed to vary to quantify the TFA-dependent pseudo first-order loss of MVKOO. The unimolecular decay only contributes to the intercept of the *k*_pseudo_ vs [TFA] (TFA concentration) plot and does not influence the slope, which yields the bimolecular rate coefficient.

**Figure 3:**
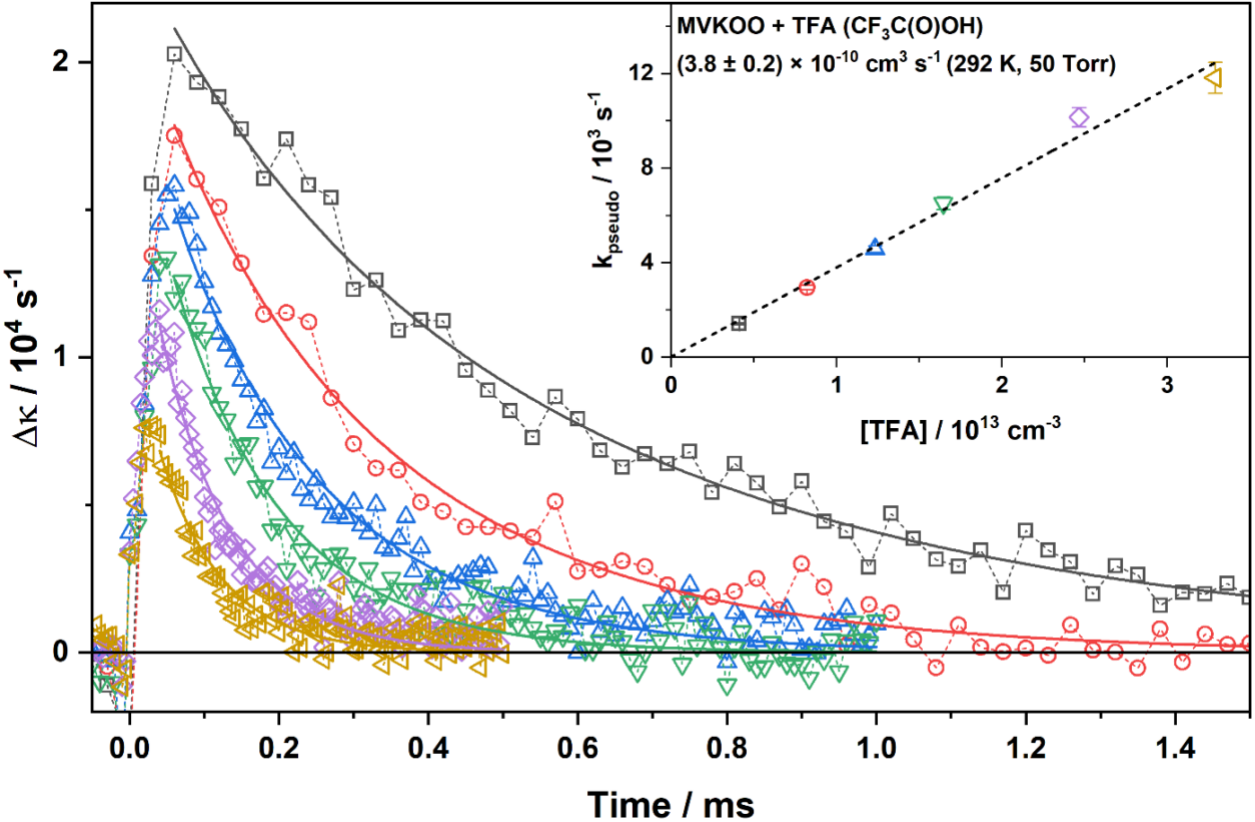
Kinetic decay traces for the MVKOO + TFA (CF_3_C­(O)­OH) reaction obtained at 50 Torr total pressure and 292 K. The solid lines correspond to fits of the experimental decay profiles using fit function E1, which accounts for simultaneous first- and second-order loss processes. The self-reaction rate coefficient was fixed at *k*′_SR_ = 3.6 × 10^7^ cm s^–1^. The inset shows the derived pseudo-first-order rate coefficients plotted as a function of TFA concentration, from which the linear fit slope provides the bimolecular rate coefficient for the MVKOO + TFA reaction. The intercept from the linear fit of *k*_pseudo_ vs [TFA] (∼20 ± 400 s^–1^) was near zero, with large uncertainty reflecting *k*_pseudo_ dominates the fit.

The intercepts from the linear fit of *k*_pseudo_ vs [TFA] plots were not constrained, and the values obtained were consistent with *k*_uni_, ranging from 20–1000 s^–1^ in the pressure and temperature-dependent measurements. Fixing the intercept at *k*_uni_ = 50 s^–1^ had no impact on the derived bimolecular rates, as the unconstrained intercept values were already close to this value or within the uncertainty of fit. The influence of the fixed *k*′_SR_ value on the derived bimolecular rate coefficient was also evaluated by varying *k*′_SR_ over its uncertainty lower limit (1.8 × 10^7^ cm s^–1^) to upper limit (5.1 × 10^7^ cm s^–1^), and no significant change in the extracted bimolecular rate coefficient was observed (Figure S8). This analysis highlights that the bimolecular rate coefficient is largely insensitive to the fixed *k’*_SR_.

The reaction of MVKOO with TFA was measured at 50 Torr and 292 K, as shown in Figure 3, yielding a bimolecular rate coefficient of (3.8 ± 0.2) × 10^–10^ cm^3^ s^–1^. To assess potential pressure dependence, measurements were extended over the 50–125 Torr range at 292 K (Figure S9). No significant pressure dependence was observed within this range, and an average rate coefficient of *k*_TFA_ (292 K) = (3.8 ± 0.3) × 10^–10^ cm^3^ s^–1^ was obtained from the pressure-dependent data. These results indicate that the MVKOO + TFA reaction proceeds in the high-pressure limit under the present experimental conditions. The measured rate coefficient for MVKOO + TFA is comparable in magnitude to those reported for other Criegee intermediates reacting with TFA.^16^ In particular, CH_2_OO reacts with TFA with a nearly identical reaction rate coefficient at 294 K, while (CH_3_)_2_COO exhibits slightly faster kinetics. The comparable reactivity across these stabilized Criegee intermediates reflects a similar reaction pathway for MVKOO reaction with TFA.

### Predictive SAR Model

To further rationalize the observed kinetics in terms of molecular structure, the reaction of MVKOO with TFA was examined within the context of a structure–activity relationship framework. Predictive models such as those developed by Chhantyal-Pun et al. relate bimolecular rate coefficients for Criegee intermediate reactions to the dipole moments of the reactants.^17^ In this approach, the dominant interaction for acid insertion is treated as a dipole–dipole capture between the Criegee intermediate and the acidic proton donor. The SAR model expresses the bimolecular rate coefficient as a function of the reduced mass (μ) and the dipole moments of the two reactants (μ_D1_, μ_D2_), with *k* = μ^–0.5^ ((1.9 ± 0.2) × 10^–21^ (μ_D1_ × μ_D2_)^2/3^ – (6.3 ± 0.7) × 10^–21^), incorporating the dipole–dipole capture contribution.^17^

The SAR predicted rate coefficient for MVKOO reaction with FA and TFA are (1.9 ± 0.2) × 10^–10^ cm^3^ s^–1^ and (3.8 ± 0.4) × 10^–10^ cm^3^ s^–1^, respectively. These values are in good agreement with the measured rate coefficients, demonstrating that the SAR model accurately represents the reaction mechanism. These reactions exhibit rapid reaction rates that are largely dictated by the dipole–dipole attraction of the interacting species. The measured MVKOO + TFA reaction exceeds the collision limit (3.2 × 10^–10^ cm^3^ s^–1^) and approaches the dipole-capture limit (7.4 × 10^–10^ cm^3^ s^–1^).

### Temperature Dependence of MVKOO + TFA Kinetics

The reactions of the Criegee intermediate with acids proceed via the formation of a prereaction complex (PRC). The temperature dependence in reactions involving a PRC arises because the formation and dissociation of the PRC introduce an additional energy barrier that can influence the observed rate. In such cases, the overall bimolecular rate is not solely determined by direct reaction but also by the stability of the PRC involved, which is inherently temperature-dependent. Figure 4 shows the temperature dependence of the measured rate coefficient, *k*_TFA_(T), for the MVKOO + TFA reaction. The representative decay traces used to extract these values are shown in Figure S10 of the Supporting Information. The obtained temperature-dependent *k*_TFA_(T) values were fitted using the kinetic model proposed by Chhantyal-Pun et al. for halogenated acids, which explicitly accounts for the formation of an intermediate PRC along the reaction pathway.^16^

E2 $$k\left(T\right)=AT^{2}\exp\left(\frac{\Delta H}{\mathrm{RT}}\right)+k_{r}$$

**Figure 4:**
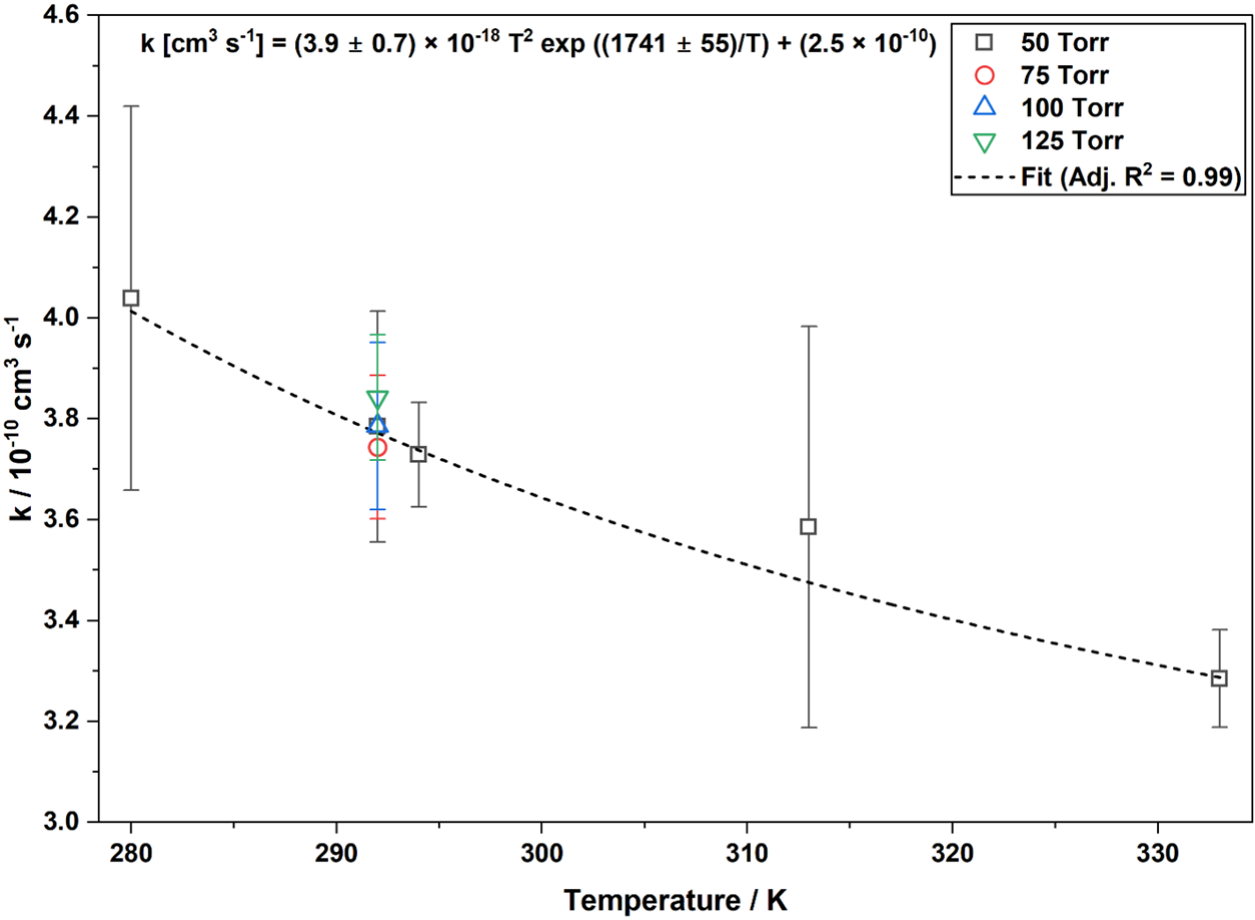
Temperature dependence of the measured rate coefficients *k*_TFA_ for the MVKOO + TFA reaction. The dashed line represents the fit to E2.

Here, Δ*H* represents the difference between the activation enthalpy for PRC dissociation back to reactants and that for crossing the transition state to products, while *k*_r_ denotes the rate coefficient for the direct reaction without involving the PRC.^16^ In the initial fit step, the values of A, Δ*H*/*R*, and *k*_r_ were varied to obtain the best fit values for each. Subsequently, the obtained *k*_r_ value was fixed at 2.5 × 10^–10^ cm^3^ s^–1^, and the fit was repeated.

The fit to the temperature-dependent rate coefficient values yields *A* = (3.9 ± 0.7) × 10^–18^ cm^3^ s^–1^ K^–2^ and Δ*H* = 14.5 ± 0.5 kJ mol^–1^. The positive Δ*H* indicates that the barrier for dissociation of the hydrogen-bonded PRC back to reactants is higher than the barrier along the product channel, implying that once the PRC is formed, it preferentially evolves toward products. The measured rate coefficient varies by only ∼20% across the experimental temperature range, reflecting a weak negative temperature dependence, in line with previous reports for the CH_2_OO + TFA system.^16^ Recent computational studies for the MVKOO + FA reaction further support this observation,^8^ showing that the net insertion reaction proceeds via an effectively barrierless pathway driven by concerted H atom transfer from the acid to the Criegee intermediate and simultaneous C–O hydrogen bond formation with the Criegee carbon.

## Conclusions

The kinetics of unimolecular and bimolecular reactions of the MVKOO stabilized Criegee intermediate derived from isoprene ozonolysis have been systematically explored using cavity ring-down spectroscopy to monitor MVKOO concentrations. The bimolecular self-reaction of MVKOO is rapid, with a rate coefficient of *k*_SR_ (292 K) = (12 ± 4) × 10^–10^ cm^3^ s^–1^, although it is only significant under laboratory-scale concentrations (∼10^11^ cm^–3^). The unimolecular decay rate is estimated as *k*_uni_ (292 K) = (50  ±  21) s^–1^, consistent with previously reported experimental and computational values. Bimolecular reaction with TFA proceeds rapidly, with a rate coefficient of *k*_TFA_ (292 K) = (3.8 ± 0.3) × 10^–10^ cm^3^ s^–1^, exceeding the collision limit and approaching the dipole-capture limit, demonstrating that long-range dipole–dipole interactions dominate. A weak negative temperature dependence reflects the involvement of a prereaction complex (PRC), in agreement with mechanistic studies for similar systems.

The rate coefficient for the reaction of MVKOO with TFA is comparable to that with CH_2_OO, indicating that steric and orientational effects from the vinyl substitution counterbalance the slightly higher dipole moment of MVKOO. Predictive SAR modeling accurately reproduces the observed rate coefficients for both MVKOO + TFA and MVKOO + FA reactions, supporting the conclusion that these reactions are primarily driven by dipole capture. Overall, these findings contribute to a better understanding of the atmospheric relevance of Criegee intermediate and organic acid chemistry. Such reactions produce hydroperoxy ester products in the atmosphere,^10,20^ which are proposed to undergo subsequent Criegee-mediated oligomerization, contributing to the growth of low-volatility species and secondary organic aerosol formation. The rapid reaction of TFA with MVKOO indicates that this chemistry would act as a significant atmospheric sink, limiting the tropospheric lifetime of TFA to just a few days in regions with abundant Criegee intermediates.

## Supplementary Material



## Notes

Experimental methods and characterization of (*Z*/*E*)-1,3-diiodobut-2-ene by ^1^H NMR and UV–Vis spectroscopy; correction and assignment of residual interfering signals; precursor concentration dependent kinetics; pressure- and temperature- dependent kinetics of MVKOO self-reaction and reactions with trifluoroacetic acid, SO_2_, and formic acid; computational dipole moments and SAR calculations; comparison of observed and calculated rate coefficients for Criegee intermediates; all associated figures (S1–S11), tables (S1–S2), and references (PDF)
