# Supplementary material for: Temperature-Dependent Kinetics of the Methyl Vinyl Ketone Oxide Criegee Intermediate: Self-Reaction and Reaction with Trifluoroacetic Acid
Source: J Phys Chem A. 2026 May 27;130(23):4411–20. doi: 10.1021/acs.jpca.6c01738 (PMC13267087; doi:10.1021/acs.jpca.6c01738)
Supplement: Supplementary file 1 [file jp6c01738_si_001.pdf]

Supporting Information

Temperature-Dependent Kinetics of the Methyl Vinyl Ketone Oxide Criegee Intermediate: Self-Reaction and Reaction with Trifluoroacetic Acid

Saurabh Khodia,* Eddie Reilly, Maria de los Angeles Garavagno, and Andrew J. Orr-Ewing

School of Chemistry, University of Bristol, Cantock's Close, Bristol BS8 1TS, UK.

* Author for correspondence: s.khodia@bristol.ac.uk

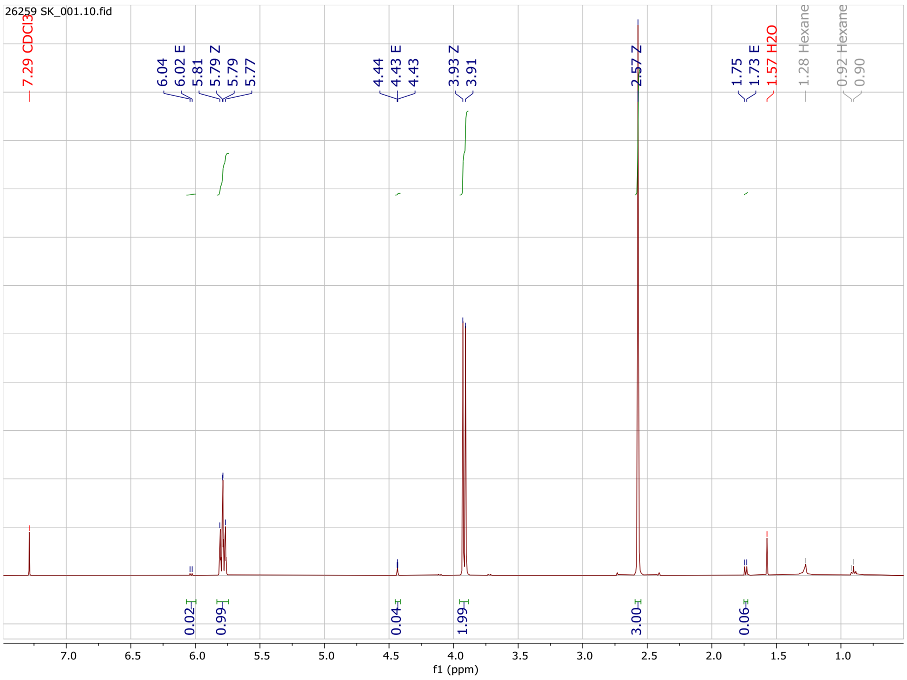

Figure S1. 400 MHz ^{1}H NMR of (Z)-1,3-diiodobut-2-ene at 298 K. The trace amount of hexane is removed by using a freeze-pump-thaw cycle before use.

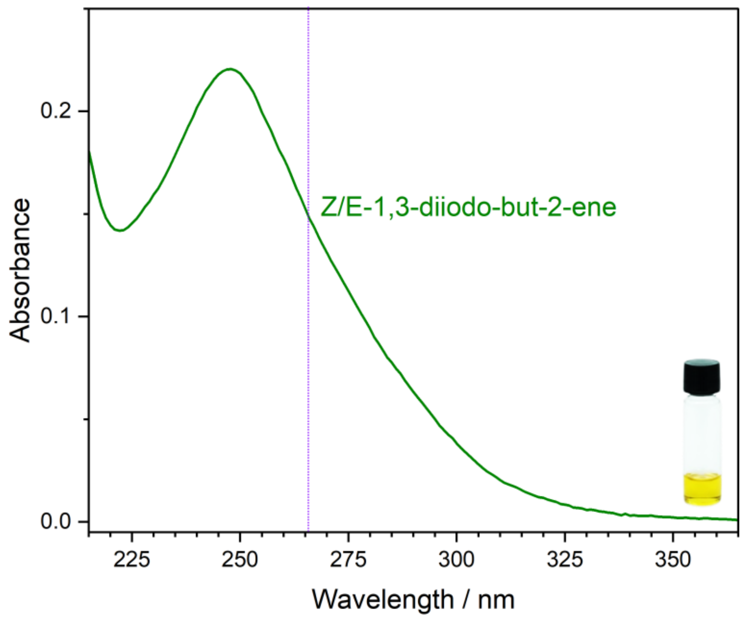

Figure S2. UV-Vis absorption spectrum of (Z/E)-1,3-diiodobut-2-ene in hexane. The vertical dashed purple line represents the 266 nm wavelength used for photolysis of (Z/E)-1,3-diiodobut-2-ene in the CRDS experiments.

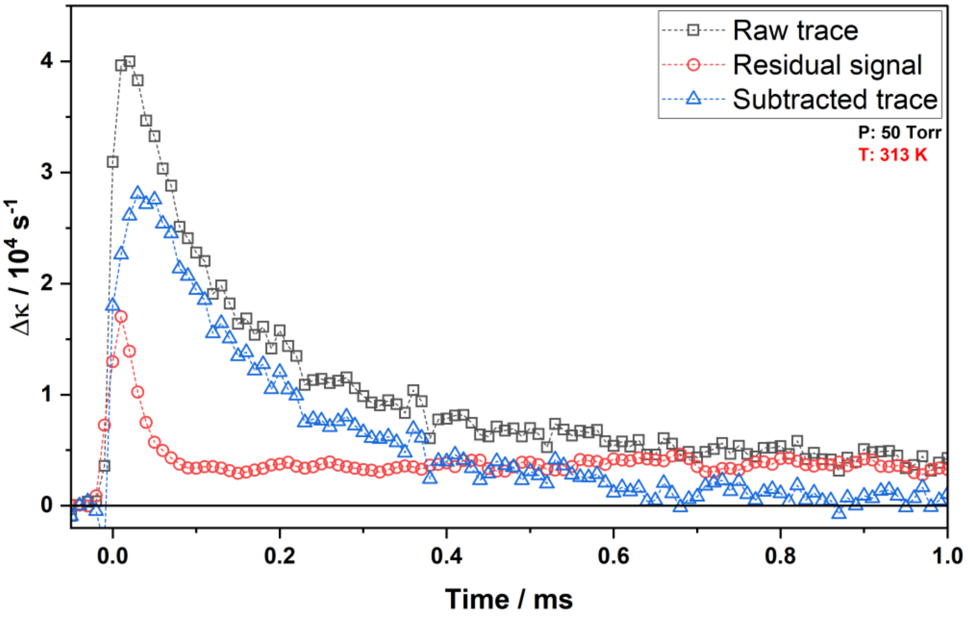

Figure S3. Example correction of residual signal in the bimolecular kinetics of MVKOO. Black squares represent the raw signal for the reaction of MVKOO + TFA (1.5 × 10^{13} cm^{-3}) at 50 torr total pressure and a temperature of 313 K. Red circles show the signal obtained with excess TFA (>5 × 10^{14} molecule cm^{-3}), which selectively scavenges MVKOO and isolates the residual interference signal at the probe wavelength (360nm). Blue triangles denote the interference corrected MVKOO decay obtained by subtracting the red circles from the black squares.

Assignment of probable interfering species in MVKOO decay at probe wavelength 360 nm

A residual absorption signal is observed at the probe wavelength of 360 nm even in the presence of excess TFA (Figure S3, red circles). This signal reflects contributions from species other than MVKOO, including primary photoproducts and secondary iodine-mediated byproducts during MVKOO generation. The residual signal remains essentially unchanged at excess TFA concentrations ($>5 \times 10^{14}$ molecule cm^{-3}) and does not decrease with further increases in TFA, confirming that it originates from species that are not efficiently scavenged by TFA.

The temporal profile of the residual signal exhibits two distinct components. An initial rapid rise followed by decay with a time constant of ~30 µs is observed, occurring on the same timescale as MVKOO generation from 1,3-diiodobut-2-ene photolysis in excess oxygen. A slowly decaying baseline then persists over the 10 ms experimental window. The following possibilities are considered to explain the residual signal, based on their formation pathways and expected absorption at the probe wavelength:

1. **Iodoperoxy adduct (IMVKOO):** These species form directly following photolysis of 1,3-diiodobut-2-ene, where the resulting monoiodoalkene ($\cdot C_4H_6I$) radical reacts with O_2, producing IMVKOO. The initially formed excited adduct ($IMVKOO^{\#}$) can either dissociate to produce MVKOO + I or undergo collisional stabilization by a third body (M) to form the IMVKOO peroxy radical.

   $$ICH_3C{=}CHCH_2I + h\nu\ (266\ nm) \rightarrow (\cdot C_4H_6I) + I$$
   $$(\cdot C_4H_6I) + O_2 \leftrightharpoons IMVKOO^{\#}$$
   $$IMVKOO^{\#} \rightarrow MVKOO + I$$
   $$\mathbf{IMVKOO^{\#} + M \rightarrow IMVKOO + M}$$

   Analogous to ICH_2OO, which exhibits broad absorption across 250–400 nm,[1] IMVKOO as a primary photoproduct may absorb at the probe wavelength, potentially contributing to the early-time residual signal.

2. **Iodine monoxide (IO):** IO can form through multiple reaction pathways during MVKOO generation. Proposed routes involve reactions of MVKOO with I atoms (MVKOO + I → IO + MVKO) or IMVKOO with I atoms (IMVKOO + I → IO + IMVKO). Secondary channels involve photodissociation of MVKOO to produce $O(^1D)$, followed by collisional relaxation to $O(^3P)$ and subsequent reaction leading to IO. However, this pathway requires multiple intermediate steps and would also rely on excitation at 360 nm to generate $O(^1D)$. Given the relatively low intensity of the probe laser at this wavelength in our CRDS experiments, efficient production of $O(^1D)$ via this route is unlikely. IO has known broad absorption in the 350–450 nm range,[2] making it a plausible contributor to the residual signal at 360 nm. The IO contribution is expected to become more apparent at longer times due to formation via secondary reaction pathways

3. **MVKOO reaction products:** Products from MVKOO self-reactions or its reaction with acid or iodine may absorb at 360 nm. For example, MVKOO + I → MVKO + IO produces methyl vinyl ketone (MVKO), which absorbs in the 250–400 nm range.[3] However, all such reactions require MVKOO as a precursor. Under conditions of excess TFA, MVKOO

is scavenged, which prevents secondary product formation and effectively eliminates any related interference.

4. **Free iodine atoms (I):** I atoms are produced immediately upon 266 nm photolysis of the diiodo precursor. While I atoms themselves have no absorption at 360 nm, the precursor species and other photofragments can produce I-mediated byproducts that may overlap the probe region, particularly at later times.

The early rise and decay of the residual signal are consistent with IMVKOO, which follows formation kinetics similar to MVKOO (Scheme 1, main text). The fast decay of the residual signal occurs within the MVKOO formation timescale (~200 µs), supporting this assignment. The observed ~30 µs decay time constant indicates rapid dissociation of IMVKOO adducts to MVKOO. Under the experimental conditions, IMVKOO can undergo self-reaction and reaction with MVKOO. However, the $IMVKOO^{\#}$ → MVKOO + I pathway seems to dominate because dissociation is more facile in the absence of efficient third-body stabilization. Collisional stabilization of IMVKOO will enable IMVKOO + MVKOO reactions, which would manifest as a pressure dependence in the MVKOO self-reaction rate coefficient k_{SR}. The absence of a strong pressure dependence in k_{SR} indicates that stabilized IMVKOO contributes only minimally and that rapid dissociation to MVKOO is the dominant pathway.

The long-lived residual signal is best accounted for by IO formation through pathways independent of MVKOO concentration. MVKOO is efficiently scavenged by excess TFA, precluding MVKOO-mediated IO production. Instead, IO can form via I + O reactions, where an oxygen atom is generated through two-photon absorption of the 266 nm photolysis laser (2 × 4.66 eV), possibly resonance enhanced at the one-photon level, which can access the repulsive $B^3\Sigma_u^-$ state of O_2, leading to dissociation and formation of O atoms. This mechanism provides a plausible explanation for the long-lived signal, but further studies are needed to clarify the underlying IO formation pathways.

Overall, all measured MVKOO decay traces were corrected for residual absorption from interfering species at the probe wavelength. Any first-order loss of MVKOO due to these species would appear as an intercept in the bimolecular kinetics, such as in the linear fit of k_{pseudo} vs [TFA]. The observed intercepts were consistent with k_{uni} and showed no significant deviation, signifying minimal to no impact from these interferences on the measured bimolecular rates.

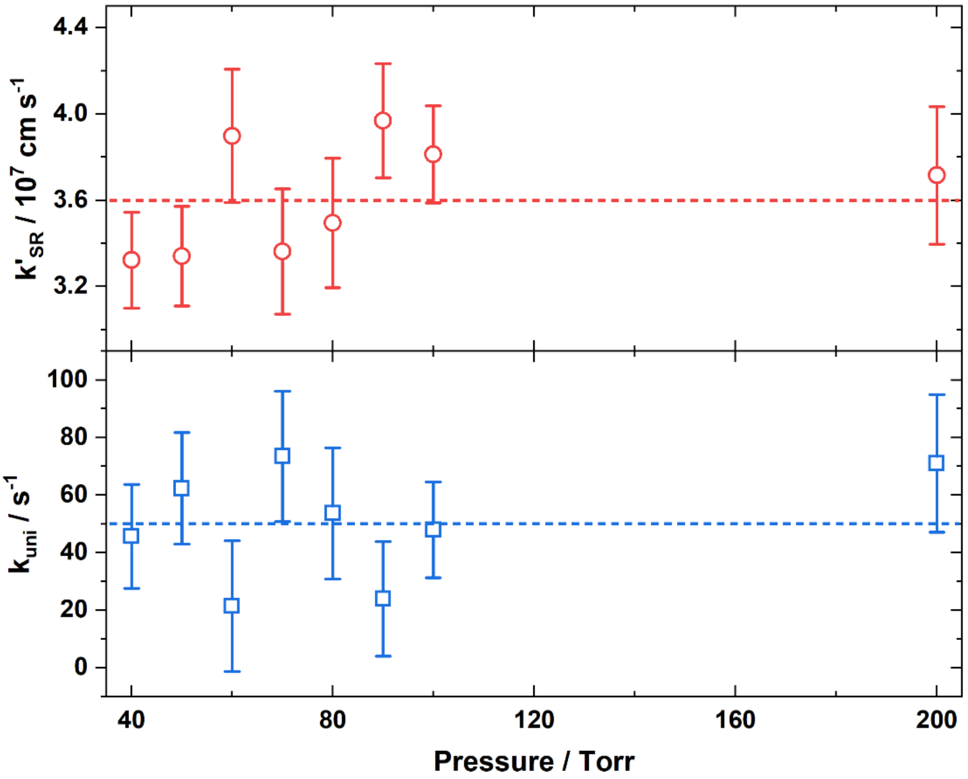

Figure S4. Tests of the pressure dependence of the self-reaction k'_{SR} and unimolecular k_{uni} rate coefficients for MVKOO in the absence of co-reactant. Error bars represent the 1σ uncertainty from the fit function E1 applied to the MVKOO decay traces. All measurements were made at 292 K.

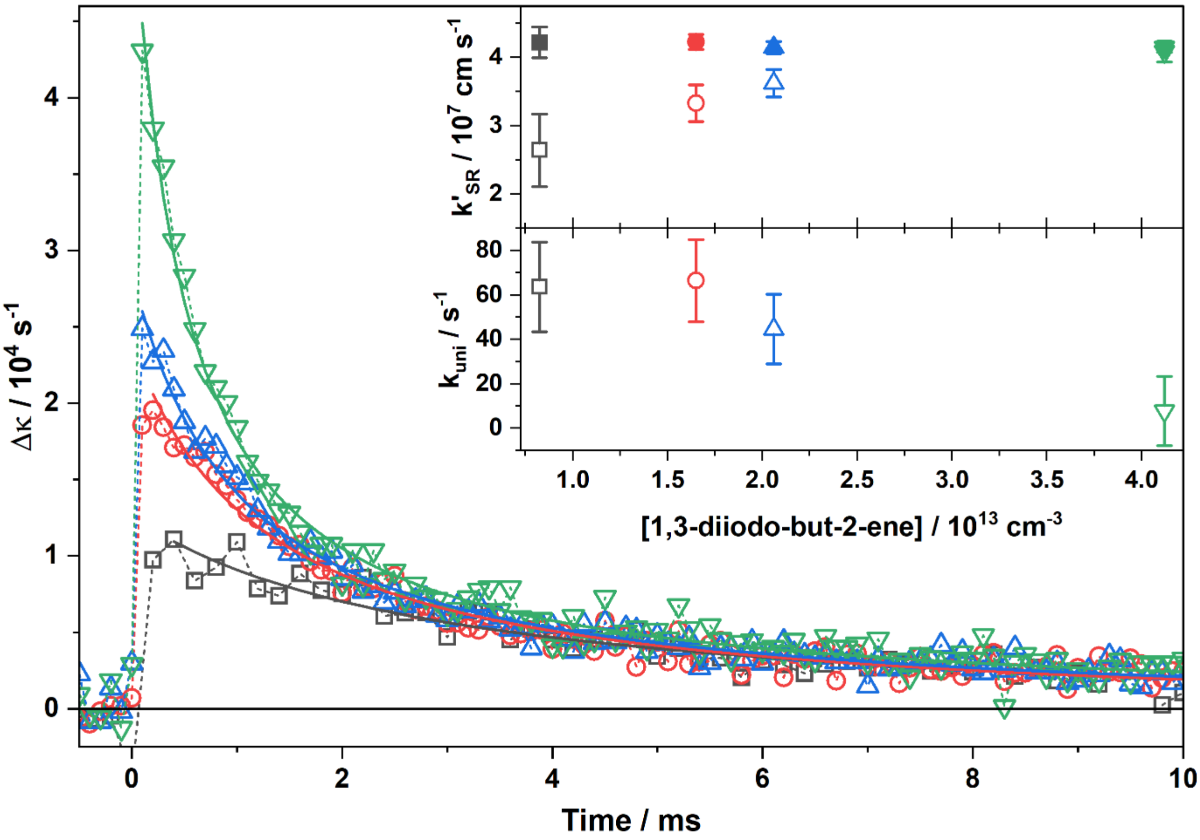

Figure S5. Pseudo-first-order and second-order reaction kinetics of MVKOO in the absence of co-reactant. The left plot shows MVKOO decay traces obtained at different concentrations of the (Z/E)-1,3-diiodobut-2-ene precursor. The solid lines show the fits obtained using the simultaneous first- and second-order decay fit function (E1). The inset plot shows first- (k_{uni}) and second-order (k'_{SR}) rate coefficients obtained from the fits (the latter scaled by the absorption cross section at 360 nm), at respective precursor concentration. The solid symbols (top inset) show k'_{SR} from a fit considering only second-order kinetics versus the simultaneous first- and second-order fit (hollow symbols). All measurements were made at a total pressure of 50 Torr and at 292 K.

Table S1. Summary of the experimental conditions and simultaneous first- (k_{uni}) and second-order (k'_{SR}) fit values for the MVKOO decay in the absence of co-reactant. The O_2 concentration was maintained at $[O_2]$ = (3.3 – 4.1) × 10^{17} cm^{-3} and the bath gas was N_2.

**Precursor 1,3-diiodobut-2-ene (13DI) concentration dependence study (Total pressure 50 Torr and temperature 292 K)**				
**[13DI]**	[MVKOO]	[MVKOO]/[13DI]	k_{uni}	k'_{SR}
**/ 10^{13} cm^{-3}**	/ 10^{11} cm^{-3}	% yield	/ s^{-1}	/ 10^7 cm s^{-1}
**0.83**	1.7	2.1	(64±20)	(2.6±0.5)
**1.7**	3.3	2.0	(66±18)	(3.3±0.3)
**2.1**	3.9	1.9	(45±16)	(3.6±0.2)
**4.1**	7.4	1.8	(8±16)	(4.1±0.2)
Average			**(46±18)**	**(3.4±0.6)**
**Pressure dependence study (temperature 292 K and [13DI] = 2.7 × 10^{13} cm^{-3})**				
**Pressure**			k_{uni}	k'_{SR}
**/ Torr**			/ s^{-1}	/ 10^7 cm s^{-1}
**40**			(46±18)	(3.3±0.2)
**50**			(62±19)	(3.3±0.2)
**60**			(21±23)	(3.9±0.3)
**70**			(73±23)	(3.4±0.3)
**80**			(54±23)	(3.5±0.3)
**90**			(24±20)	(4.0±0.3)
**100**			(48±17)	(3.8±0.2)
**200**			(71±24)	(3.7±0.3)
Average			**(50±21)**	**(3.6±0.8)**
**Temperature dependence study (Pressure 50 Torr and [13DI] ~ 0.9 × 10^{13} cm^{-3}**				
**Temperature**			k_{uni}	k'_{SR}
**/ K**			/ s^{-1}	/ 10^7 cm s^{-1}
**273**			(52±13)	(2.9±0.5)
**294**			(92±16)	(2.2±0.4)
**318**			(199±22)	(2.0±0.5)
**323**			(270±38)	(3.4±0.8)
Average				**(2.6±1.1)**

Table S2. Comparison of the observed and calculated self-reaction and unimolecular reaction rate coefficient values for CH_2OO, $(CH_3)_2COO$ and MVKOO Criegee intermediates. The collisional and dipole capture limits were calculated for MVKOO using the expressions proposed by Chhantyal-Pun et al.[4] Values in bold are from this work.

sCI	*d** (Å)	*μ** (D)	$k_{collision}$ / 10^{-10} cm^3 s^{-1}	$k_{capture}$ / 10^{-10} cm^3 s^{-1}	k_{SR} / 10^{-10} cm^3 s^{-1}	k_{uni} / s^{-1}
MVKOO	**5.7**	**5.27**	**2.8**	**17.6**	**12 ± 4**	**50 ± 21**
$(CH_3)_2COO$	**4.4**	**5.42**	1.7[4]	19.8[4]	6 ± 1[4]	305 ± 70[4]
CH_2OO	**3.2**	**4.39**	1.2[4]	20.8[4]	0.74 ± 0.06[5]	11.6 ± 8.0[5]

**d*: maximum molecular diameter of the Criegee intermediates (distance from terminal O to farthest H atom)
**μ*: Dipole moments calculated at the DFT-D3(BJ)/B3LYP/aug-cc-pVTZ level of theory (Figure S11)

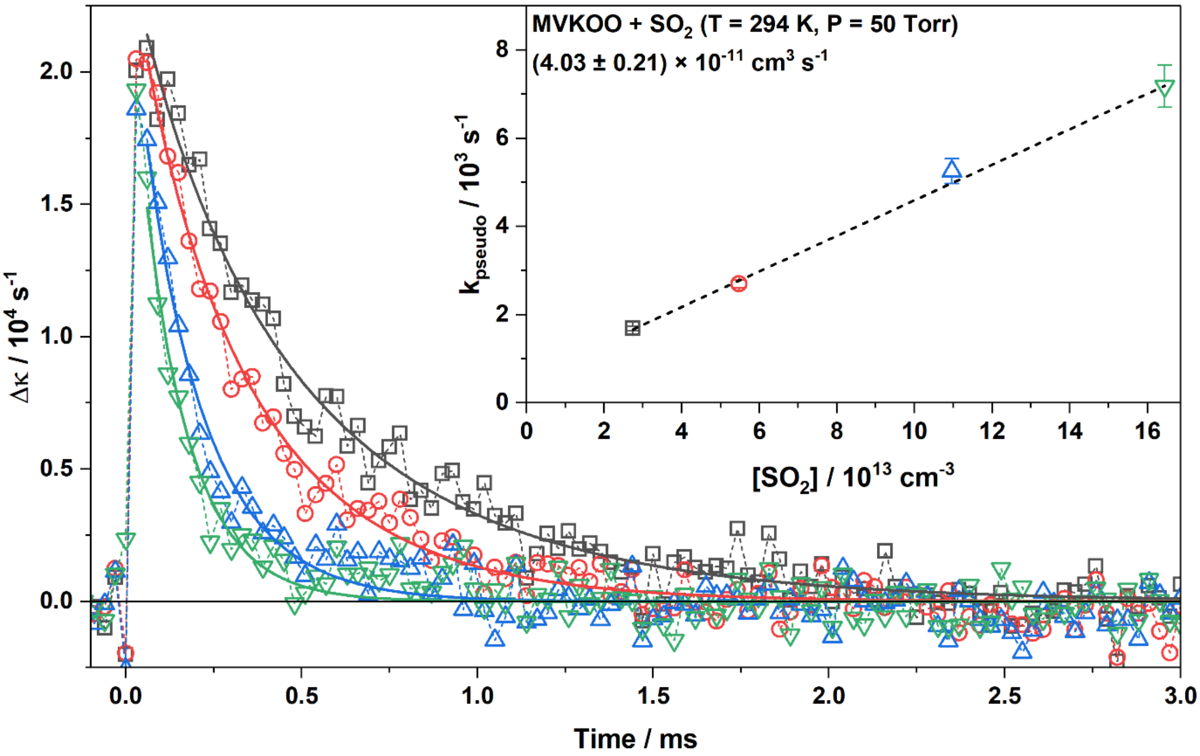

Figure S6. Kinetic plots for the reaction of MVKOO + SO_2 at 50 torr total pressure and a temperature of 294 K. The solid lines show fits to the experimental data points obtained using the fit function (E1). The inset shows the pseudo-first-order decay rate coefficients plotted as a function of [SO_2] concentration. The solid line in the inset plot is a linear fit from which the bimolecular rate coefficient is obtained. In fit function E1, k'_{SR} was fixed at 3.6×10^7 cm s^{-1}.

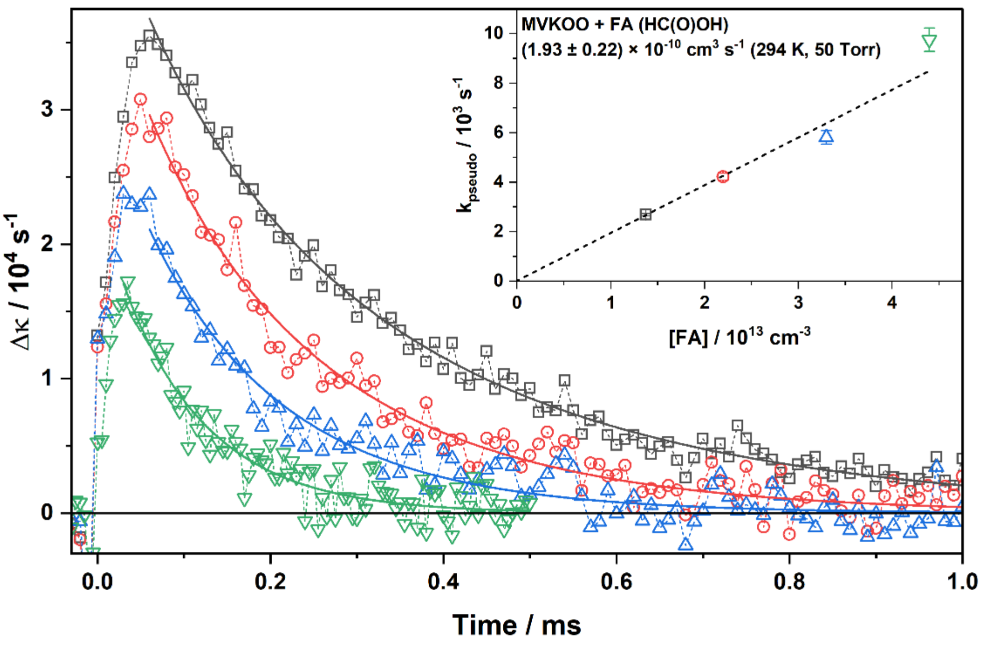

Figure S7 Kinetic plots for the reaction of MVKOO + FA (HC(O)OH) at 50 torr total pressure and a temperature of 294 K. The solid lines show fits to the experimental data points obtained using the fit function (E1). The inset shows the pseudo-first-order decay rate coefficients plotted as a function of [FA] concentration. The solid line in the inset plot is a linear fit from which the bimolecular rate coefficient is obtained. In fit function E1, k'_{SR} was fixed at 3.6 × 10^7 cm s^{-1}. The intercept of the linear fit of k_{pseudo} vs [FA] (~30 ± 400 s^{-1}) was consistent with k_{uni} for MVKOO.

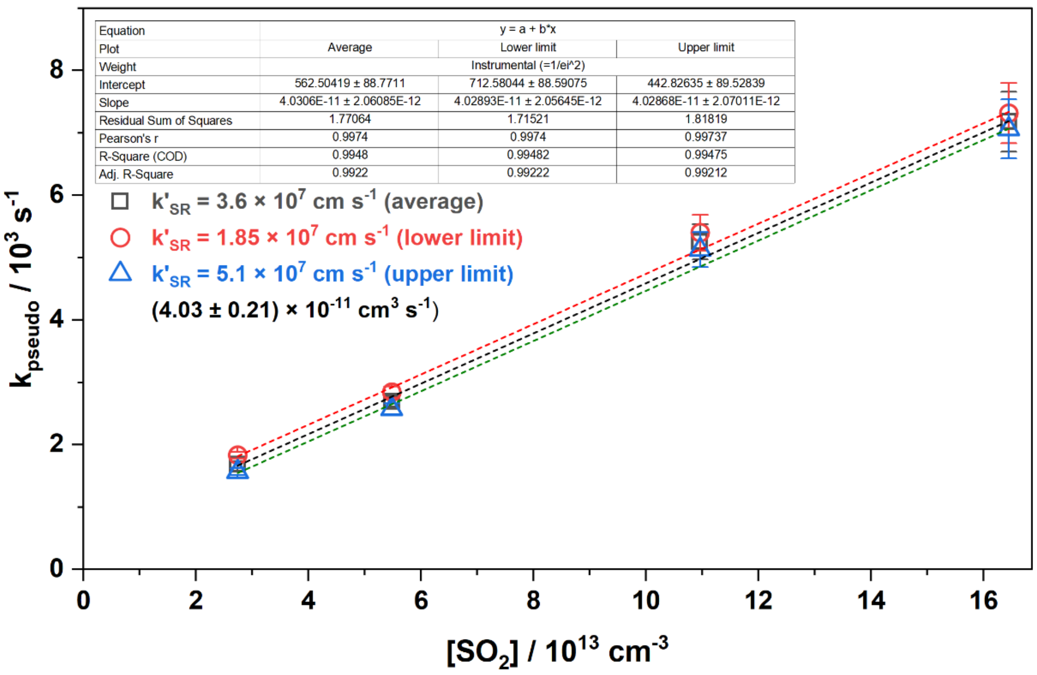

Equation	y = a + b*x		
Plot	Average	Lower limit	Upper limit
Weight	Instrumental (=1/ei^2)		
Intercept	562.50419 ± 88.7711	712.58044 ± 88.59075	442.82635 ± 89.52839
Slope	4.0306E-11 ± 2.06085E-12	4.02893E-11 ± 2.05645E-12	4.02868E-11 ± 2.07011E-12
Residual Sum of Squares	1.77064	1.71521	1.81819
Pearson's r	0.9974	0.9974	0.99737
R-Square (COD)	0.9948	0.99482	0.99475
Adj. R-Square	0.9922	0.99222	0.99212

Figure S8. Pseudo-first order decay coefficient for MVKOO as a function of [SO_2] measured at 50 Torr and 294 K. The bimolecular rate coefficient was determined using three different k'_{SR} values in equation (E1), showing no significant influence on the results.

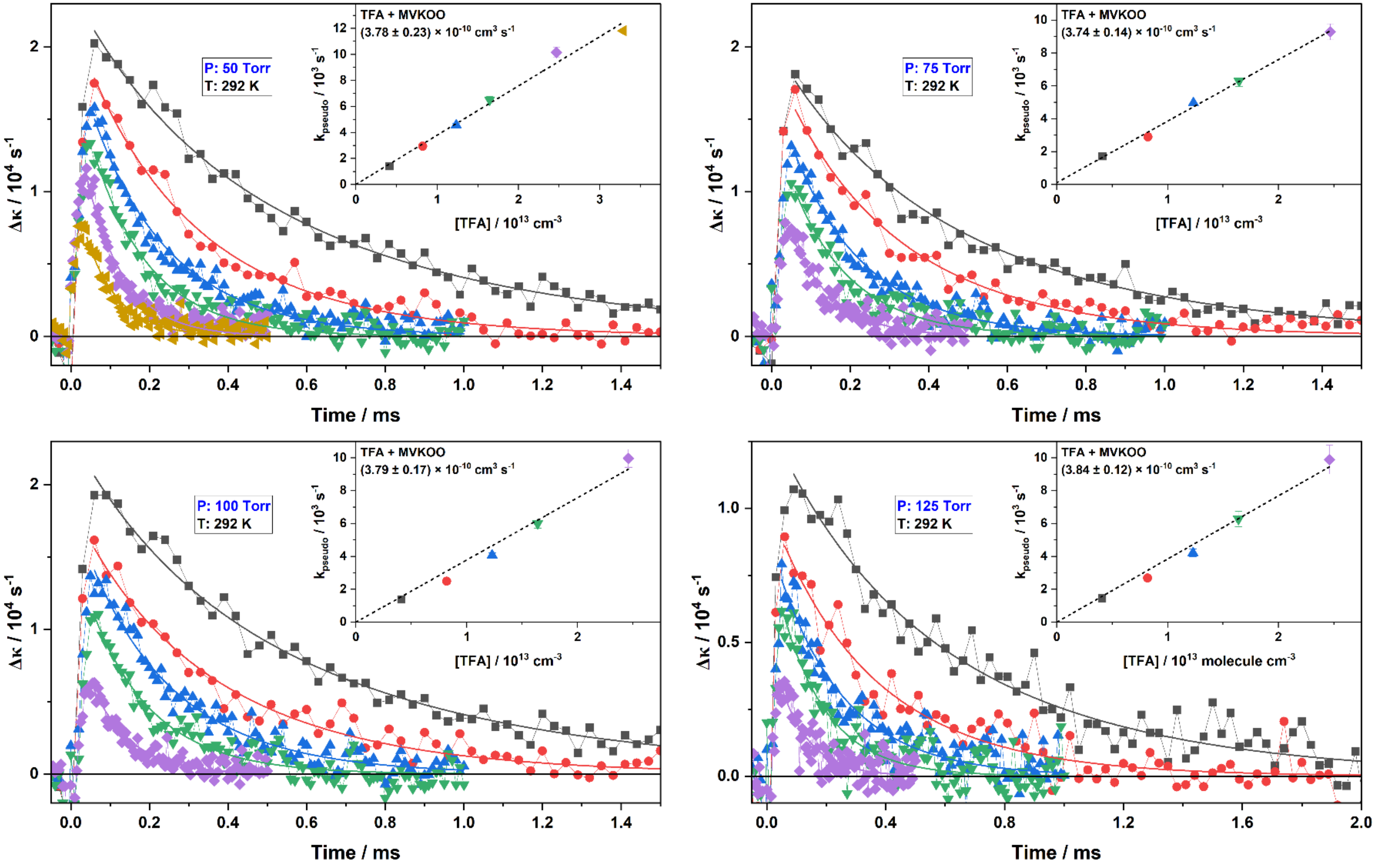

Figure S9. Pressure-dependent kinetic decay traces for the MVKOO + TFA ($CF_3C(O)OH$) reaction obtained at 292 K and different pressures in the range from 50 to 125 Torr. The solid lines correspond to fits of the experimental decay profiles using fit function E1, which accounts for simultaneous first- and second-order loss processes. The self-reaction rate coefficient was fixed at $k'_{SR} = 3.6 \times 10^{7}$ cm s^{-1}. The insets show the derived pseudo-first-order rate coefficients plotted as a function of TFA concentration, from which the linear fit slopes provide the bimolecular rate coefficients for the MVKOO + TFA reaction.

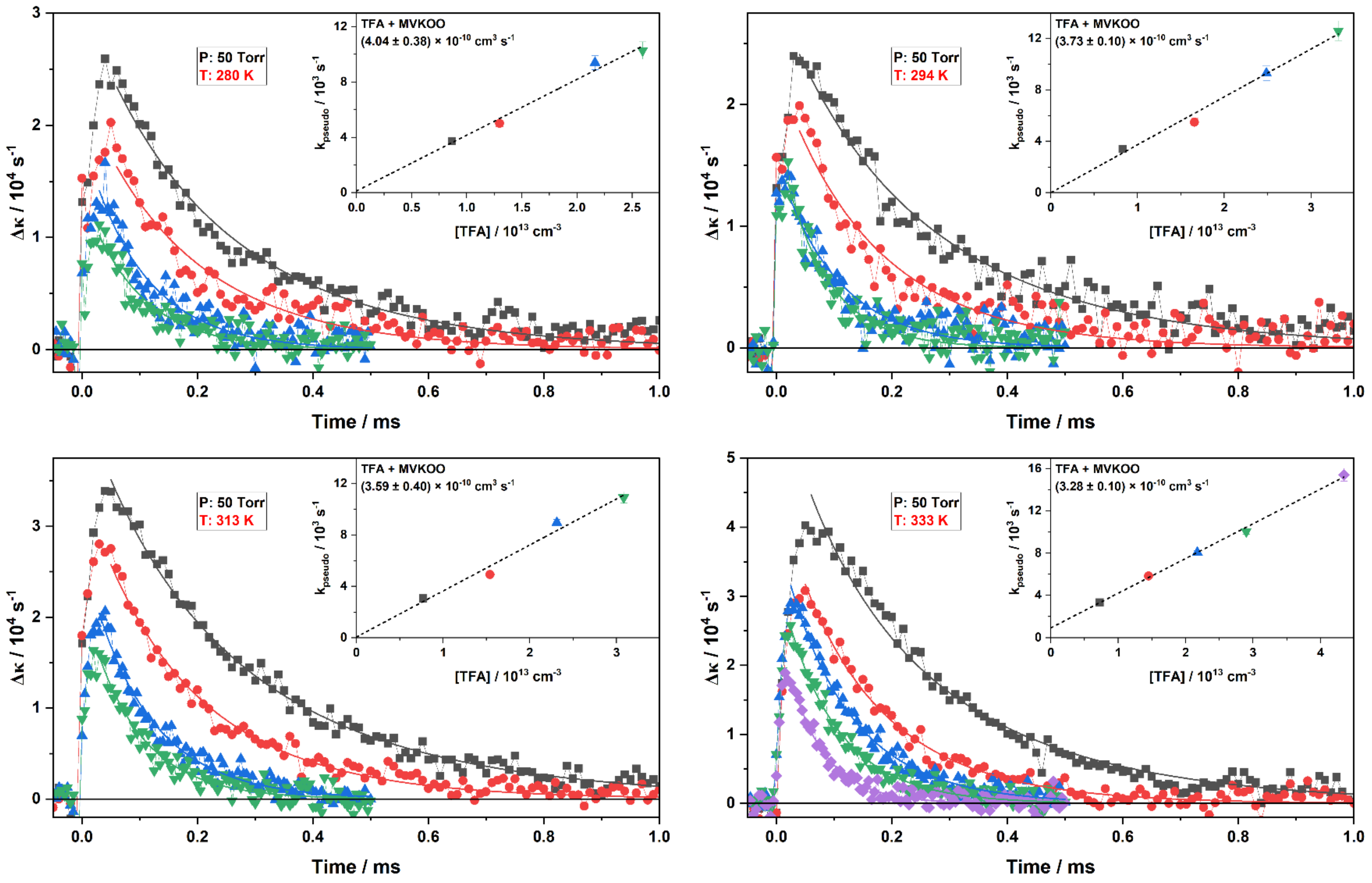

Figure S10. Temperature-dependent kinetic decay traces for the MVKOO + TFA ($CF_3C(O)OH$) reaction obtained at 50 Torr and different temperatures in the range from 280 to 333 K. The solid lines correspond to fits of the experimental decay profiles using fit function E1, which accounts for simultaneous first- and second-order loss processes. The self-reaction rate coefficient was fixed at $k'_{SR} = 3.6 \times 10^7$ cm s^{-1} as it shows no significant temperature dependence (Figure 2, main text). The insets show the derived pseudo-first-order rate coefficients plotted as a function of TFA concentration, from which the linear fit slopes provide the bimolecular rate coefficients for the MVKOO + TFA reaction.

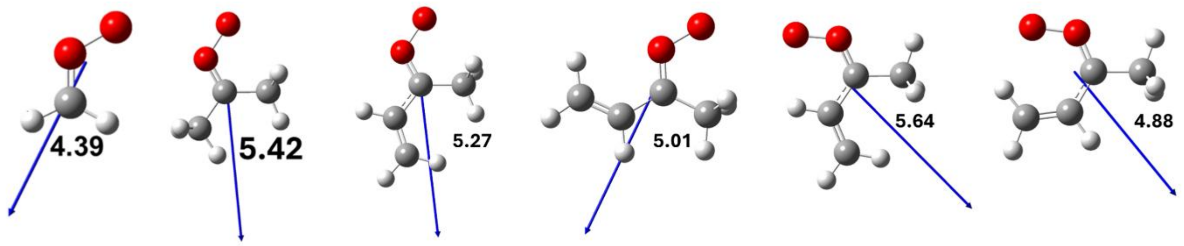

Figure S11. Dipole moments calculated at the DFT-D3(BJ)/B3LYP/aug-cc-pVTZ level of theory. CH_2OO 4.39 D, $(CH_3)_2COO$ 5.42 D, and MVKOO exhibits conformer-dependent values of 5.27 D (syn–trans), 5.01 D (syn–cis), 5.64 D (anti–trans), and 4.88 D (anti–cis). The conformer-averaged dipole moment of MVKOO (5.2 D) was used in the SAR model to estimate reaction rate coefficients with trifluoroacetic acid (2.23 D)[6] and formic acid (1.55 D).[6]

References

(1) Gravestock, T. J.; Blitz, M. A.; Bloss, W. J.; Heard, D. E. A Multidimensional Study of the Reaction CH_2I+O_2: Products and Atmospheric Implications. *ChemPhysChem* **2010**, *11* (18), 3928-3941. DOI: 10.1002/cphc.201000575.

(2) Spietz, P.; Gómez Martín, J. C.; Burrows, J. P. Spectroscopic studies of the I_2/O_3 photochemistry: Part 2. Improved spectra of iodine oxides and analysis of the IO absorption spectrum. *J. Photochem. Photobiol. A Chem.* **2005**, *176* (1), 50-67. DOI: 10.1016/j.jphotochem.2005.08.023.

(3) Gierczak, T.; Burkholder, J. B.; Talukdar, R. K.; Mellouki, A.; Barone, S. B.; Ravishankara, A. R. Atmospheric fate of methyl vinyl ketone and methacrolein. *J. Photochem. Photobiol. A Chem.* **1997**, *110* (1), 1-10. DOI: 10.1016/S1010-6030(97)00159-7.

(4) Chhantyal-Pun, R.; Welz, O.; Savee, J. D.; Eskola, A. J.; Lee, E. P. F.; Blacker, L.; Hill, H. R.; Ashcroft, M.; Khan, M. A. H.; Lloyd-Jones, G. C.; Evans, L.; Rotavera, B.; Huang, H.; Osborn, D. L.; Mok, D. K. W.; Dyke, J. M.; Shallcross, D. E.; Percival, C. J.; Orr-Ewing, A. J.; Taatjes, C. A. Direct Measurements of Unimolecular and Bimolecular Reaction Kinetics of the Criegee Intermediate $(CH_3)_2COO$. *J. Phys. Chem. A* **2017**, *121* (1), 4-15. DOI: 10.1021/acs.jpca.6b07810.

(5) Chhantyal-Pun, R.; Davey, A.; Shallcross, D. E.; Percival, C. J.; Orr-Ewing, A. J. A kinetic study of the CH_2OO Criegee intermediate self-reaction, reaction with SO_2 and unimolecular reaction using cavity ring-down spectroscopy. *Phys. Chem. Chem. Phys.* **2015**, *17* (5), 3617-3626. DOI: 10.1039/C4CP04198D.

(6) Chhantyal-Pun, R.; Rotavera, B.; McGillen, M. R.; Khan, M. A. H.; Eskola, A. J.; Caravan, R. L.; Blacker, L.; Tew, D. P.; Osborn, D. L.; Percival, C. J.; Taatjes, C. A.; Shallcross, D. E.; Orr-Ewing, A. J. Criegee Intermediate Reactions with Carboxylic Acids: A Potential Source of Secondary Organic Aerosol in the Atmosphere. *ACS Earth Space Chem.* **2018**, *2* (8), 833-842. DOI: 10.1021/acsearthspacechem.8b00069.
